# Hyperglycaemia results from beta-cell dysfunction in critically ill children with respiratory and cardiovascular failure: a prospective observational study

**DOI:** 10.1186/cc7732

**Published:** 2009-02-26

**Authors:** Catherine M Preissig, Mark R Rigby

**Affiliations:** 1 Emory University School of Medicine, Department of Pediatrics, Division of Pediatric Critical Care Medicine, Children's Healthcare of Atlanta at Egleston, 1405 Clifton Road, Atlanta, GA 30322, USA

## Abstract

**Introduction:**

Hyperglycaemia is common in critical illness and associated with poor outcome. Glycaemic control using insulin may decrease morbidity and mortality. Many questions remain about the cause of critical illness hyperglycaemia (CIH). Our objective was to investigate the endocrinological basis of paediatric CIH.

**Methods:**

C-peptide and blood glucose (BG) levels were assessed in 41 children aged 2 to 18 years old who were admitted to our paediatric intensive care unit (PICU). Patients who developed CIH, defined as persistent BG above 7.7 mmol/L, were treated with insulin infusion to achieve BG levels between 4.4 and 7.7 mmol/L. C-peptide levels were compared with respect to CIH development and degree of organ failure in all patients. Respiratory and cardiovascular failure were defined as need for mechanical ventilation and need for vasoactive infusions, respectively. Clinical and laboratory parameters, including c-peptide levels, were assessed.

**Results:**

Of 41 children enrolled, 18 had respiratory failure only, 11 had both respiratory and cardiovascular failure, and 12 had neither respiratory or cardiovascular failure. Nine patients with respiratory failure only, 10 with both respiratory and cardiovascular failure, and none with no respiratory or cardiovascular failure developed CIH. Patients with CIH and respiratory and cardiovascular failure (n = 10) had very low c-peptide levels (4.4 ng/mL) despite significantly elevated mean BG levels (10.8 mmol/L), while those with CIH and respiratory failure only had very high c-peptide levels (11.5 ng/mL) with mean BG of 9.9 mmol/L. Low endogenous insulin production in those with respiratory and cardiovascular failure was associated with rapid onset of CIH, illness severity, higher insulin requirement and longer mechanical ventilation days, PICU length of stay and CIH duration.

**Conclusions:**

Primary beta-cell dysfunction as defined by low endogenous c-peptide production appears to be prevalent in critically ill children with both respiratory and cardiovascular failure who develop CIH, whereas elevated insulin resistance appears to be the prominent cause of CIH in children with respiratory failure only. Our finding that beta-cell dysfunction is present in a subset of critically ill children with CIH challenges the assertion from adult studies that CIH is primarily the result of elevated insulin resistance.

## Introduction

Over the past several years critical illness hyperglycaemia (CIH) and glycaemic control have emerged as prominent issues in critical care [1-14]. In addition to determining the impact of hyperglycaemia and glycaemic control on patient outcome, many questions remain regarding CIH, including a clear understanding of its basic pathogenesis. Persistent hyperglycaemia of any aetiology represents a state of metabolic dysregulation resulting from an imbalance of insulin production and insulin sensitivity in target tissues. Type 1 and 2 diabetes mellitus (DM) represent extremes of this aetiological spectrum in chronic disease. Although both have similar clinical phenotypes and adverse sequelae, they have different aetiologies – autoimmune-induced beta-cell destruction in type 1 DM versus peripheral insulin resistance in type 2 DM. Understanding the aetiology of DM significantly impacts disease course and therapeutic approach.

CIH is often considered an extreme form of 'stress' hyperglycaemia resulting from a surge of endogenous counter-regulatory hormones, but other diabetogenic factors are likely to contribute to CIH, and differentiate it from a pure sympathoadrenal 'fight or flight' response [1,4-6]. Critical illness is associated not only with increased endogenous counter-regulatory hormones, but with pro-inflammatory mediators, oxidative stress and therapeutic interventions, all of which interfere with insulin receptor signalling and/or insulin-regulated glucose channels, and directly interfere with proper glucose transport and utilisation in peripheral cells [6,15-27].

CIH is prevalent in paediatric intensive care units (PICUs) and is an independent risk factor for morbidity and mortality [28-38]. We routinely screen for and treat hyperglycaemia with insulin in our PICU, and have reported that about 20% of all our admissions develop CIH [35]. In comparing our approach in children with published adult studies, we find substantial differences in glycaemic management, including higher insulin requirements and shorter duration of treatment in children [35]. These disparities led us to question whether there were other differences between paediatric and adult CIH such as metabolic aetiology. The objective of this study was to investigate the endocrinological aetiology of CIH in subsets of children with critical illness and determine clinical factors associated with this condition. Herein we report that primary beta-cell dysfunction and resultant absolute insulin deficiency may contribute to the development of CIH in select critically ill children, contrasting the current dogma in adults that CIH is primarily due to elevations in resistance of peripheral tissues to insulin.

## Materials and methods

### Study site

The PICU at the Children's Healthcare of Atlanta at Egleston is a quaternary 30-bed multidisciplinary unit with high acuity medical and surgical conditions that cares for patients from infancy to 21 years of age.

### CIH – definition, assessment and management

Our standard care physician-initiated, nurse-driven protocol was used to screen for and treat CIH in our PICU [35]. Patients with respiratory failure requiring mechanical ventilation and those with cardiovascular failure requiring vasoactive infusions are considered at high risk for hyperglycaemia in our PICU and are routinely screened for CIH by twice daily bedside glucometry (Accucheck Inform, Baltimore, MD, USA). Patients younger than six months old, weighing less than 5 kg, or with hepatic failure or type 1 DM are excluded from CIH screening and treatment. CIH is defined as a blood glucose (BG) level above 7.7 mmol/L on two occasions one to two hours apart, and infused insulin (Novalin R, Princeton, NJ, USA) is automatically initiated and titrated to achieve a BG level of 4.4 and 7.7 mmol/L via our nurse-driven algorithm in patients with CIH.

### Research design and patient selection

An Institutional Review Board-approved prospective observational study was conducted and consent was obtained for all patients before study enrollment. Patients admitted to our PICU aged 2 to 18 years old without severe hepatic insufficiency or failure, known DM or requiring continuous renal replacement therapy were eligible for enrollment. Patients admitted to our PICU were screened by study staff for possible inclusion into the study, and those meeting our inclusion criteria were enrolled after informed consent was obtained. Patients for consideration were those admitted sequentially to our PICU, and only those that did not meet enrollment criteria or did not consent to enrollment were excluded. We enrolled 12 patients without respiratory or cardiovascular failure, and 29 patients with respiratory failure and/or cardiovascular failure. Serum samples from all participants were analysed for BG and c-peptide levels (ARUP laboratories; Salt Lake City, Utah, USA), where control fasting c-peptide levels are reported to be 0.8 to 4 ng/mL. In patients who developed CIH, samples were drawn after CIH diagnosis but before initiation of exogenous insulin treatment. In those at high risk for hyperglycaemia who did not develop CIH, samples were drawn within 48 hours of intubation or initiation of vasoactive infusions. In those without respiratory or cardiovascular failure, samples were drawn within 48 hours of PICU admission.

### Demographic and clinical data

Baseline characteristics and other clinical information were obtained on all patients. Illness severity and organ dysfunction were quantified using paediatric logistic organ dysfunction (PELOD) scoring [39]. We adapted and expanded the vasopressor score devised by Hatherill and colleagues to quantify vasopressors/inotrope use [40]. Scores were additive and assigned as follows: 1 = dopamine less than 10 μg/kg/minute; 2 = dopamine 10 μg/kg/minute or above; 2 = noradrenaline or adrenaline less than 0.5 μg/kg/minute; 3 = noradrenaline or adrenaline 0.5 μcg/kg/minute or above; 1 = milrinone less than 0.5 μg/kg/minute; 2 = milrinone 0.5 μg/kg/minute or above; 3 = vasopressin less than 4 mU/kg/minute; 4 = vasopressin 4 mU/kg/minute or above. When available, creatinine levels drawn as part of routine management were evaluated. Levels were available in 6 of 12 patients not at high risk for CIH, and were available for analysis in all patients at high risk for CIH. All creatinine levels were drawn between 12 hours before or after c-peptide levels were drawn.

Caloric delivery and make-up was evaluated for all patients with respiratory failure at the time BG and c-peptide levels were drawn. Because most patients without respiratory failure were on oral feeds, detailed caloric data was not available. Caloric goals were determined by Schoefield and White formulas for all patients by a certified PICU nutritionist.

### Statistical analysis

BG and c-peptide levels were compared using Student's two-tailed *t *tests, where a p < 0.05 was considered statistically significant. Other results in different groups were compared either by Student's *t *test for normally distributed data, Mann-Whitney U tests for non-normally distributed data, or chi squared tests for comparison of proportions. Statistical testing was performed using SPSS 15.0, (Chicago, IL, USA).

## Results

### Patient baseline characteristics

We assessed BG and c-peptide levels in 12 PICU patients without respiratory or cardiovascular failure, 18 patients with respiratory failure only, and 11 patients with respiratory and cardiovascular failure. No patient without respiratory or cardiovascular failure developed CIH. Patients with respiratory and/or cardiovascular failure were split into two groups: those who did not develop CIH (persistent BG of more than 7.7 mmol/L) and those that did develop CIH. For those with respiratory failure, only 9 of the 18 developed CIH, and 10 of the 11 with both respiratory and cardiovascular failure developed CIH. No significant differences in age, gender or ethnicity were apparent between any groups (Table 1). No patient was hyperglycaemic requiring insulin at PICU discharge. No patient had clinical or laboratory evidence of renal failure or chronic DM (Table 1).

**Table 1:** Baseline characteristics of all groups included in the study

	**Patients with CIH**		**Patients without CIH**	
	
	**Respiratory failure only** **(n = 9)**	**Respiratory and CV failure** **(n = 10)**	**No organ failure** **(n = 12)**	**Respiratory failure only** **(n = 9)**
**Male** **gender**	67% (6)	50% (5)	67% (8)	55% (5)
**Mean age (years)**	8.4 (6 to 12)	10 (3 to 18)	6.2 (2–12)	7.8 (4 to 14)
**Ethnicity**	Cauc = 5 AA = 3 Hisp = 1	Cauc = 7 AA = 3 Hisp = 0	Cauc = 8 AA = 3 Hisp = 1	Cauc = 6 AA = 2 Hisp = 1
**Mean PICU** **LOS (days)**	11.4 (5 to 14)	13.1 (10 to 17)	3.8 (2 to 6)	5.2 (3 to 7)
**Mean MV days**	7 (4 to 12)	8.3 (6 to 14)	0	3.2 (1 to 6)
**Mean PELOD score** **at study entry**	11.4 (1 to 21)	21.5 (11 to 30)	1.8 (0 to 10)	5.4 (1 to 11)
**IV steroids**	44% (4)	100% (10)	50% (6)	44% (4)
**Average creatinine level** **(mg/dL)**	0.5 (0.4 to 0.9)	0.7 (0.4 to 1.7)	0.4 (0.3 to 0.7)	0.4 (0.3 to 0.8)
**PICU survival**	100% (9)	90% (9)	100% (12)	100% (9)
**Patients requiring insulin at PICU discharge**	0% (0)	0% (0)	0% (0)	0% (0)

There were no significant differences in gender, age, ethnicity or creatinine levels in any group. CIH patients with respiratory failure and cardiovascular failure had significantly higher PELOD scores and mean PICU LOS compared with all other groups. AA = African American; Cauc = caucasian; CIH = critical illness hyperglycaemia; CV = cardiovascular; Hisp = Hispanic; IV = intravenous; LOS = length of stay; MV = mechanical ventilation; PELOD = paediatric logistic organ dysfunction score; PICU = paediatric intensive care unit.

Results are shown as means, with ranges in parentheses.

In general, patients with CIH had significantly higher PICU lengths of stay, mechanical ventilation days and PELOD scores compared with those without CIH (Table 1). In patients with CIH, those with both respiratory and cardiovascular failure had longer PICU lengths of stay (13.1 days), mechanical ventilation days (8.3 days) and PELOD scores (21.5) compared with those with CIH with respiratory failure only (11.4 days, 7 days and 11.4, respectively) (Table 1).

### BG and c-peptide levels

**Figure 1:**
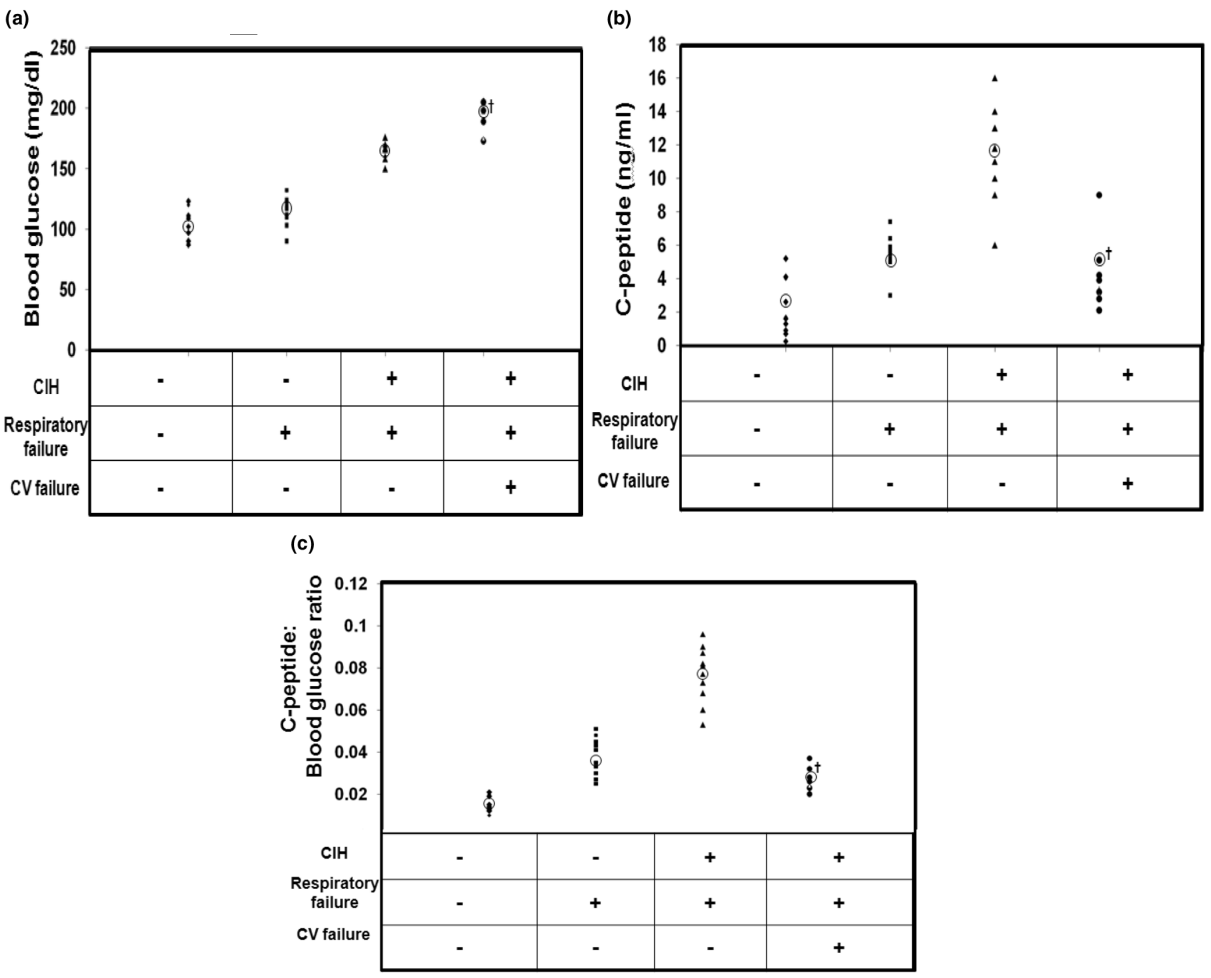
Blood glucose levels, C-peptide levels and c-peptide:blood glucose ratios in all patients. **(a)** Blood glucose levels, **(b)** c-peptide levels and **(c)** c-peptide:blood glucose ratios in all patients. Circled characters denote means for particular groups. Critical illness hyperglycaemia (CIH) patients with respiratory and cardiovascular (CV) failure had significantly higher blood glucose levels but significantly lower c-peptide levels compared with those with CIH with respiratory failure only († p < 0.05). Patients without any organ failure, those with respiratory failure without CIH and those with respiratory failure with CIH had c-peptide:blood glucose ratios that increased linearly. Patients with CIH with respiratory and cardiovascular failure had a drastic decline in c-peptide:blood glucose ratio, reflecting that this analysis assumes functional beta-cells able to generate more endogenous insulin for greater degree of hyperglycaemia.

Blood glucose and c-peptide levels in patients without respiratory or cardiovascular failure, and those in patients with respiratory failure but without CIH were not statistically different (5.8 mmol/L versus 6.1 mmol/L, and 2.3 ng/mL versus 5.3 nl/mL, respectively; Figures 1a and 1b). In patients that developed CIH, BG and c-peptide levels were drawn at time of CIH diagnosis. Although CIH patients with both respiratory and cardiovascular failure had higher BG levels compared with CIH patients with respiratory failure only (10.8 mmol/L versus 9.9 mmol/L; p < 0.05), they had significantly lower c-peptide levels (4.4 ng/mL versus 11.5 ng/mL; p < 0.05; Figures 1a and 1b).

### C-peptide: blood glucose ratios

In patients with functional beta-cells, increased insulin resistance correlates with higher c-peptide:BG ratios. Patients without respiratory or cardiovascular failure, those with respiratory failure without CIH, and those with respiratory failure and CIH had a linear increase in c-peptide:BG ratios (0.015, 0.038 and 0.08, respectively) consistent with increasing elevations in insulin resistance (Figure 1c). Patients with CIH with respiratory and cardiovascular failure had significantly lower c-peptide:BG ratio compared with those with CIH and respiratory failure only (p < 0.05), highlighting the functional deficiency of beta-cells in these patients during hyperglycaemia (Figure 1c).

### CIH duration and severity

All patients with CIH were treated with exogenous insulin after CIH diagnosis until resolution. Patients with respiratory and cardiovascular failure developed CIH on average on PICU day 0.7 versus PICU day 3.1 for those with respiratory failure only (p < 0.05), almost five times more rapidly (Table 2). Those with respiratory and cardiovascular failure were hyperglycaemic 60% longer than those with respiratory failure only, 8.7 versus 5.8 days (p < 0.05), and had higher peak insulin requirements (0.19 U/kg/hour versus 0.13 U/kg/hour; Table 2). All patients with CIH either started their hospital admission in the PICU or were transferred to the PICU within 24 hours of hospital admission.

**Table 2:** Characteristics of critical illness hyperglycaemia

	**Respiratory failure only** **(n = 9)**	**Respiratory and CV failure** **(n = 10)**
	
**Mean CIH days**	5.8 (2 to 12)	8.7 (5 to 13)*
**Mean days to CIH development**	3.1(0.25 to 6)	0.7 (0 to 3)*
**Insulin requirement 24 hours after CIH diagnosis (U/kg/hour)**	0.1 (0.05 to 0.12)	0.12 (0.05 to 0.16)
**Peak insulin requirement (U/kg/hour)**	0.13 (0.06 to 0.2)	0.19 (0.16 to 0.2)*
**24-hour insulin dose:** **C-peptide ratio**	0.008 (0.005 to 0.04)	0.02 (0.015 to 0.07)*

Patients with cardiorespiratory failure have more severe CIH compared with those with respiratory failure only. Ranges are in parentheses.

CIH = critical illness hyperglycaemia; CV = cardiovascular.

Asterisks show p < 0.05 compared to respiratory failure only group.

### Nutritional considerations

**Figure 2:**
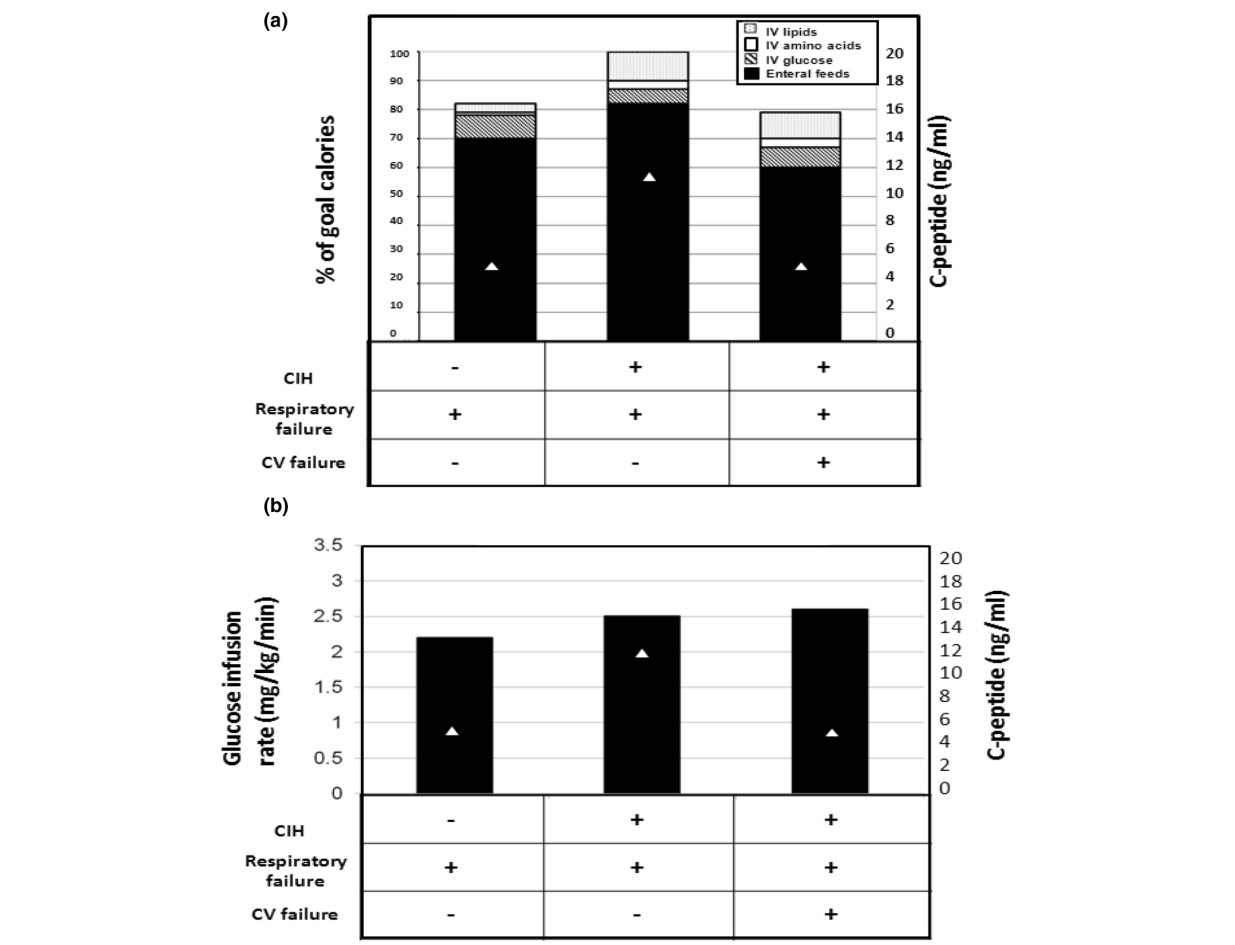
Caloric goals, make-up and glucose infusion rates in patients with respiratory and/or cardiovascular failure. **(a) **Caloric goals, make-up and **(b)** glucose infusion rates in patients with respiratory and/or cardiovascular (CV) failure. Percentage and make-up of caloric delivery did not differ significantly in patients in with or without critical illness hyperglycaemia (CIH). Because endogenous insulin and c-peptide production can be specifically related to glucose infusion rates, it is notable that glucose infusion rates in these groups and subgroups did not differ significantly either.

In an attempt to prevent or treat hyperglycaemia in critical care settings some practitioners withhold nutrition. Additionally, some CIH studies in adults have been criticised because of the concern that proactive nutritional supplementation 'uncovers' CIH [1,2]. The stress of critical illness substantially increases metabolic demands and, further, children have up to three to four times higher relative basal metabolic needs than adults. We therefore did not attempt to prevent CIH or modify BG levels by adjusting nutrition in our PICU. Because BG levels and endogenous insulin production are related to exogenous calorie and glucose input, we assessed patients for total caloric delivery and intravenous glucose infusion rates (GIR) at the time BG and c-peptide levels were drawn. Patients without respiratory or cardiovascular failure could not be assessed for specific caloric intake as all were receiving an *ad libitum *age-specific oral diet, and those on intravenous fluids were receiving no more than 5% dextrose at maintenance rates. Precise supplementation was determined in all patients with respiratory and/or cardiovascular failure, as all received a combination of quantifiable enteral (via nasogastric tube) and intravenous supplementation. There was no difference in total calories delivered, caloric composition or GIR in any group with respiratory and/or cardiovascular failure, with or without CIH, suggesting against a critical role of the amount or type of calorie delivery in the development of CIH (Figures 2a and 2b).

## Discussion

Consistent with our previous work, about two-thirds of children in our PICU requiring mechanical ventilation developed CIH, and more than 90% of patients with both respiratory and cardiovascular failure developed CIH [35]. Patients with respiratory and cardiovascular failure with CIH had evidence of severe primary beta-cell dysfunction, as evidenced by very low c-peptide levels in the face of significant hyperglycaemia. This finding supports the proposition that many factors commonly elevated in critical illness, including pro-inflammatory cytokines, catecholamines and glucocorticoids, may directly suppress beta-cell function and insulin secretion [4,6,15-23]. Yet severe beta-cell dysfunction did not occur in all of our patients with CIH, as those with respiratory failure only had high c-peptide levels, yet were still hyperglycaemic, and required about 0.1 U/kg/hour of exogenous insulin to maintain BG levels between 4.4 and 7.7 mmol/L. This amount of exogenous insulin is about one to two times the basal insulin requirements for euglycaemia. These patients therefore had substantial insulin resistance in the absence of absolute beta-cell dysfunction. Although insulin resistance appears to be the predominant cause of hyperglycaemia in these patients, our data does not allow the differentiation between hepatic insulin resistance and peripheral (muscle and adipose) insulin resistance. Even though these patients produce high levels of insulin, it is not enough to correct hyperglycaemia, and therefore they too display some evidence of relative beta-cell dysfunction.

C-peptide is produced on an equimolar basis with endogenous insulin, is renally excreted, and is the most reliable surrogate of endogenous insulin production in the face of normal kidney function [27]. This study excluded patients with renal failure. Only one patient in our study with CIH had evidence of mild renal insufficiency, and this was in a patient with respiratory and cardiovascular failure with low c-peptide levels. This supports our interpretations that differences in c-peptide levels were a reflection of endogenous, native beta-cell function and not an epiphenomenon of differences in renal function.

In addition to the different metabolic aetiologies of CIH in patients with both respiratory and cardiovascular failure (low c-peptide and beta-cell dysfunction) versus those with respiratory failure only (high c-peptide and insulin resistance), other important distinctions were noteworthy. Patients with respiratory and cardiovascular failure appear to have more severe CIH compared with those with respiratory failure only, as evidenced by more rapid onset of CIH, higher degree of hyperglycaemia, longer duration and higher insulin needs to restore normal glycaemic levels. By PICU discharge, CIH resolved in all patients and all were able to maintain normal glycaemic levels without exogenous insulin. Therefore, no participant had a pre-existing diagnosis or post-ICU diagnosis of DM (type 1 or type 2). None of the variables known to be associated with glucose intolerance, including older age, increased body weight and ethnicity, appeared to be associated with the aetiology of CIH in our patients [41-44]. Of caution, the sample size in this study was small, and differences may become apparent with larger studies.

It is noteworthy that certain pharmaceutical interventions commonly employed in critically ill patients can directly contribute to the development of hyperglycaemia. For example, it is well-known that catecholamines, both endogenous and exogenous, may directly suppress beta-cell function and insulin secretion [4,6,15-23]. Although we were not able to directly correlate degree of beta-cell dysfunction with any one particular vasopressor used in our patients, we were able to quantify vasopressor scores for our patients based on a modified scoring system developed by Hatherill and colleagues [40]. As described above, this scoring system is based on the number and amount of vasopressors required by a patient. We did find that vasopressor score was inversely correlated with c-peptide level, and thus beta-cell dysfunction. It will be important for future studies in this field to more specifically assess whether one particular vasopressor or inotrope may have a more significant impact on beta-cell insulin secretion compared with others, or if this dysfunction is more closely related to number of vasopressors required, or perhaps length of need for vasopressors.

Recent studies in adults suggest that CIH is primarily due to insulin resistance in the face of supra-normal beta-cell function [19,20,24-27]. Yet studies from the 1970s and 1980s suggest that in times of severe stress or trauma, such as that during severe (i.e. military) trauma or sepsis, beta-cell dysfunction is present in hyperglycaemic patients [45-47]. A case series from 2006 of children with meningococcal disease fits with our more generalisable observations [48]. In this report, children with meningococcal sepsis had higher peak BG levels and low insulin levels compared with children with meningococcal bacteraemia without sepsis. The authors suggest that the hyperglycaemia and beta-cell dysfunction they observed was due to a unique attribute of severe *Neisseria meningitidis *disease [48]. Our data indicate that the contribution of beta-cell dysfunction to hyperglycaemia in patients with severe illness is not a rare or disease-specific phenomenon. At least in children, this is not a condition limited to meningococcal disease, severe trauma or sepsis, and can occur in those suffering from severe critical illness due to many conditions.

Our findings suggest that CIH can be categorised based on clinical and aetiological factors. For example, CIH in children occurs rapidly and is associated with low c-peptide levels, suggestive of beta-cell dysfunction; or more gradually and is associated with increased c-peptide levels suggestive of insulin resistance. In either case children require substantial amounts of exogenous insulin to achieve normal glycaemic levels, and CIH resolves with resolution of critical illness. These characteristics differ substantially compared with adult CIH. Of note, most adult CIH studies evaluate patients aged about 70 years old, essentially representing findings in a geriatric population [1,2,11,14,27]. Adult, or perhaps more aptly, senescent CIH develops rapidly, primarily results from elevated insulin resistance, requires less insulin for glycaemic control and resolves more gradually [1,2,27]. Further studies are needed to determine the aetiological cause of CIH in younger and middle-aged adults.

Many factors in acute illness may cause beta-cell dysfunction, including elevations in pro-inflammatory cytokines, catacholamines and glucocorticoids [1,4,6,15-23,48-51]. We hypothesise that beta-cells, known to be exquisitely sensitive to rapid physiological changes, may become dysfunctional if these changes acutely occur above a certain threshold. These same changes occurring more gradually may allow beta-cells to adapt and function at supraphysiological levels over time. It may also require a fixed time for changes to occur in target tissues which result in such elevated insulin resistance to cause hyperglycaemia. Therefore, acute severe pathophysiological alterations may predispose to beta-cell dysfunction, whereas more gradual alterations may result in insulin resistance. Further, all patients with beta-cell dysfunction had cardiovascular failure requiring vasopressor infusions at the time of CIH development. Although catecholamines can contribute to both beta-cell dysfunction and insulin resistance, it may be that provision of exogenous catecholamines acutely may contribute more to beta-cell dysfunction in children.

One explanation for the development and aetiology of CIH could be differences in caloric and specifically intravenous dextrose delivery. Yet we found no significant differences in mode of caloric delivery or GIR at the time of c-peptide and insulin evaluation in our patients, nor did we find differences in those who had beta-cell dysfunction compared with those with high c-peptide levels. This suggests that the amount and type of caloric delivery was not critically important to either the development of or the metabolic cause of CIH.

Although insulin has anabolic, lipogenic and anti-inflammatory properties, some adult studies indicate the outcome benefit of glycaemic control is due to glucose normalisation rather than insulin supplementation [4,6,9,17,25,52,53]. Such conclusions may not be applicable to PICU patients, as a substantial subset of patients appear to have absolute insulin deficiency. Those children may derive clinical benefit from insulin replacement, analogous to using insulin to treat type 1 DM. Practitioners may need to rethink insulin administration and glycaemic control measures if insulin therapy may be considered as supplementing a deficient vital hormone.

Many interesting questions remain unanswered in this exciting field. In addition to further studies to elucidate more exact contribution of exogenous catecholamines to the development of hyperglycaemia, other areas for future direction include more directly discerning the contribution of insulin resistance to this pathophysiology. For example, one limitation in our study is the inability to accurately assess exact degree of insulin resistance in patients with low c-peptide levels indicative of beta-cell dysfunction. Although low c-peptide levels in these patients does indicate absolute insulin deficiency, it is likely that these patients may experience increased insulin resistance as well, even though it is likely to be to a lesser extent than those with high c-peptide levels. Future studies may employ other methods to more directly assess insulin resistance in patients, such as hyperinsulinaemic euglycaemic clamping techniques, homeostasis model assessment-estimated insulin resistance or the more recently developed quantitative insulin sensitivity check index.

## Conclusions

We describe that transient beta-cell dysfunction is a contributor to CIH in children with respiratory and cardiovascular failure, and highlight differences in CIH in children compared with adults. Understanding these differences and elucidating the pathogenesis of CIH may assist in developing individualised glycaemic goals and treatment strategies in children with life-threatening illness or injury.

## Key messages

• CIH is highly prevalent in paediatric critical illness, particularly in those with respiratory or cardiovascular failure.

• The endocrinological basis of CIH may differ in children with different disease processes, which may be different from the cause of CIH in adults with comparable disease states.

• Although CIH in adults is caused primarily by increased peripheral insulin resistance, primary beta-cell dysfunction appears to be a major cause of CIH in critically ill children with both respiratory and cardiovascular failure, whereas elevated peripheral insulin resistance appears to be the prominent cause of CIH in children with respiratory failure only.

• Understanding the aetiology of CIH may significantly impact disease course and therapeutic approach.

• Further studies are needed to discern whether treatment of CIH with insulin improves outcomes in critically ill children with peripheral insulin resistance and/or beta-cell dysfunction.

## Abbreviations

BG: blood glucose; CIH: critical illness hyperglycaemia; DM: diabetes mellitus; GIR: glucose infusion rate; PELOD: paediatric logistic organ dysfunction; PICU: paediatric intensive care unit.

## Competing interests

The authors declare that they have no competing interests.

## Authors' contributions

MRR was the primary investigator and sponsor for this study, and contributed significantly to the formulation of study design, collection of data, analysis, preparation and editing of this manuscript. CMP is the first author on the manuscript and contributed significantly to the formulation of study design, collection of data, analysis, preparation and editing of this manuscript.
